# Anti-citrullinated protein antibody positive rheumatoid arthritis is primarily determined by rheumatoid factor titre and the shared epitope rather than smoking per se

**DOI:** 10.1371/journal.pone.0180655

**Published:** 2017-07-14

**Authors:** Dan Murphy, Derek Mattey, David Hutchinson

**Affiliations:** 1 Department of Rheumatology, Royal Cornwall Hospital Trust, Truro, Cornwall, United Kingdom; 2 University of Exeter Medical School (Knowledge Spa, Cornwall Campus, Truro), Cornwall, United Kingdom; 3 Haywood Rheumatology Centre, Haywood Hospital, Stoke-on-Trent, United Kingdom; 4 Institute for Science and Technology in Medicine, Keele University, Keele, Staffordshire, United Kingdom; University of Birmingham, UNITED KINGDOM

## Abstract

**Objective:**

To analyse the relationship between rheumatoid factor (RF) titre, smoking and HLA-DRB1 alleles coding a “shared epitope” (SE) in relation to anti-citrullinated protein antibody (ACPA) positivity in rheumatoid arthritis (RA).

**Methods:**

RA patients (n = 658) attending rheumatology clinics in Cornwall, UK (cohort 1) were stratified according to RF and ACPA titre, and smoking pack years at diagnosis. A further 409 RA patients from North Staffordshire, UK (cohort 2) were studied to confirm the relationship between RF levels, smoking and ACPA positivity in relation to SE status.

**Results:**

In cohort 1 there was a trend (p<0.01) of increasing ACPA positivity rates with increasing levels of RF without statistically significant differences between patients who had never smoked and smokers (never smoked: 15/71 (21%) RF -ve, vs. 43/64 (67%) RF weak +ve, vs 88/100 (88%) RF strong +ve, ever smoked: 18/70 (26%) RF -ve vs. 66/83 (80%) RF weak +ve vs. 196/210 (93%) RF strong +ve). No significant gender difference was observed. No significant difference between smoking and ACPA positivity was seen in RF negative patients. Smoking >20 pack years conferred an increased risk of anti-CCP positive RA (158/200 (79%)), compared to having never smoked (146/235 (62%), p = <0.01), but this increased risk correlated with smokers’ RF positivity as the principal determinant on subsequent regression analysis of cohort 2. In cohort 2, ACPA positivity rates significantly increased with RF positivity and carriage of 1 or 2 SE alleles (p<0.01). Little or no relationship was observed in patients lacking SE.

**Conclusions:**

ACPA positivity in RA strongly associates with increasing RF titre independent of smoking. This relationship is dependent on carriage of SE alleles. There is no relationship between ACPA and smoking in RF negative patients.

## Introduction

Over the last 2 decades, smoking, HLA-DRB1 alleles that code a “shared epitope” (SE), and anti-citrullinated protein antibodies (ACPA) have emerged as the trinity of RA pathogenesis [1]. However, in routine clinical practice the use of both RF and ACPA remains, and for good reason: the presence of RF and ACPA in healthy individuals increases the risk of RA development over and above ACPA alone [2], and a positive RF and ACPA confers a far poorer radiological prognosis in established RA compared to either of these autoantibodies alone [3]. The 2010 ACR/EULAR RA classification criteria [4] includes a criterion that scores highly for those individuals with a strongly positive RF or ACPA (scoring 3 points), acknowledging that a strongly positive RF is of equal weighting in RA diagnosis to strongly positive ACPA. RF and ACPA co-exist in RA more than would be expected by chance alone: a study of established RA (n = 784) in Sheffield, UK, observed that 93% of RF patients were also ACPA positive [3]. The clustering of RF and ACPA is more pronounced with high titre RF as a study of 102 RA patients observed that 61% of RA patients with a RF >50 U/ml were ACPA positive (the expected frequency is 18.5% if RF>50 U/ml and ACPA occurred independently of each other) as opposed to only 25% of RA patients with a RF <50 U/ml [5]. This raises the possibility that a common process generates high titre RF and ACPA and determines a poorer prognosis in RA.

An important recent pooled analysis of 2234 RA patients casts significant doubt on the concept that smoking specifically associates with ACPA alone as RA ever smokers (n = 1318) were found to have no significant association with single ACPA positivity, (OR 0.83, 95% CI 0.56–1.24), compared to ACPA and RF double seropositivity (OR 1.4, 95% CI 1.06–1.84) [6]. Moreover, this paper included a large cohort (n = 9575) of non-RA cases. We suggest that in this non-RA cohort a much higher frequency of double autoantibody seropositivity for RF and ACPA exists than would be expected if RF and ACPA developed independently. Clustering of RF and ACPA was more pronounced in ever smokers (7 fold higher than expected) than never smokers (3 fold higher than expected), but was evident in the never smokers nonetheless. Accordingly, we have investigated this clustering of RF and ACPA in both smokers and never smokers for the first time. To date no studies have addressed if this co-existence is increased in those strongly positive for RF irrespective of smoking. Surprisingly given the obvious clinical importance ascribed to a strongly positive RF, a literature search revealed no studies investigating the relationship between the presence and levels of a positive RF, cigarette smoking, carriage of the SE alleles and the frequency of ACPA in RA. We therefore set out to answer the following two important questions: Is there a relationship between RF levels at RA diagnosis and the likelihood of testing positive for ACPA irrespective of the smoking history of the patient? Is there a relationship between the presence of the shared epitope, RF and ACPA irrespective of the smoking history of the patient?

## Methods

Two cohorts of RA patients were studied. Cohort 1 consisted of males and females attending routine rheumatology clinics at the Royal Cornwall Hospital, Cornwall, UK from February 2015 to August 2016. The data was anonymised at source, collected as part of project IRAS ID 194833, approved by South West Regional Ethical Committee. This cohort was selected to achieve an intended total cohort size of six hundred gender matched patients for analysis. Patients reviewed in clinic with a new or existing diagnosis of RA during the study period were included for analysis, with 60 patients subsequently excluded (see below). 298 male and 300 female RA patients were included to provide a suitable cohort size for serological subgroup analysis, with approximately equal numbers of each gender selected to detect potential differences in smoking rates, age of onset, and serological status rather than to represent the overall male to female prevalence ratio (Table 1). Recruitment ceased when the target cohort size was reached. Data was recorded by clinicians in a standard questionnaire via face-to-face interview. Missing data was obtained by follow up telephone conversation. Age, age of RA onset, disease duration, smoking history prior to disease development, RF levels at disease onset and subsequent ACPA levels were recorded as part of routine clinical practice. Testing for SE status does not form part of routine clinical practice and was therefore unavailable. All patients fulfilled the 2010 ACR/EULAR RA criteria at diagnosis [4].

**Table 1:** Cohort 1 demographic data.

	Males n = 298	Females n = 300
**Median age (yrs, IQR)**	64 (55–73)	64 (52–72)
**Median age at disease onset (yrs, IQR)**	54 (46–63)	54 (41–62)
**Median disease duration (yrs, IQR)**	7 (4–12)	7 (2–12)
**RF + (%)**	232/298 (78%)	225/300 (75%)
**ACPA + (%)**	217/298 (73%)	208/300 (69%)
**Mean IMD (SD)**	4.36 (1.69)	4.38 (1.79)

RF was measured with Tina-quant Rheumatoid Factors I1 Test System, by Roche Diagnostics Corporation. A value of < 14 IU/ml was considered as negative as per manufacturer guidelines. ACPA was measured by Roche Modular Analytics Second Generation E170 Anti-CCP analysis, with a negative value of <17 U/ml as per manufacturer guidelines.

As per guidelines, a negative RF or ACPA was defined as a level within the normal range, a weakly positive RF or ACPA <3 times the upper limit of normal and a strongly positive RF or ACPA >3 times the upper limit of normal [4]. Six percent (18/318) of females and 12% (40/340) of males were excluded during the collection period due to >20 years between smoking and RA diagnosis. A further 2/340 males were excluded due to incomplete data on subsequent review. Social deprivation analysis was undertaken through the UK government validated Index of Multiple Deprivation (IMD) [7], a deprivation rank score of 32,844 neighbourhoods weighted by income, health/disability, education, housing/service access, crime and living environment, converted to equal deciles.

A second cohort (n = 409) of RA patients consisted of 154 males and 255 females attending rheumatology clinics at the Haywood Rheumatology Centre in North Staffordshire, UK, in whom SE status had been determined (Table 2). Patients were recruited consecutively from a clinic established to monitor the effects of disease modifying anti-rheumatic drugs including hydroxychloroquine, sulfasalazine, D-penicillamine, gold and methotrexate. Sample collection occurred from 1994 to 2002 as part of a study to investigate the relationship between genetic factors and outcome in RA. Ethical approval was obtained from the North Staffordshire local research ethics committee and written informed consent was provided by all patients. All patients had established disease (median disease duration 9.2 years), fulfilling 1987 RA criteria at time of diagnosis [8]. A full smoking history was obtained on 386/409 patients. Smokers were defined as smoking >1 cigarette (or equivalent)/day for >1year, and categorised according to pack years smoked [9]. All patients had been genotyped for HLA-DRB1 as previously described [10].

**Table 2:** Cohort 2 demographic data.

	Males n = 154	Females n = 255
**Median age (yrs, IQR)**	59 (49–65)	58 (50–68)
**Median age at disease onset (yrs, IQR)**	49 (40–56)	48 (38–56)
**Median disease duration (yrs, IQR)**	9.0 (5–12)	9.3 (5–13)
**RF + (%)**	107/154 (69.4%)	140/255 (54.9%)
**ACPA + (%)**	124/154 (80.5%)	183/255 (71.8%)

IgM RF was measured by nephelometry at disease onset while ACPA was measured subsequently using a commercially available anti-CCP2 ELISA (Axis-Shield, Dundee, Scotland). RF levels were reported in International Units (IU). A level > 60–180 IU/ml was considered weakly positive and a level >180 IU/ml was strongly positive. ACPA measurements above 5 units/ml were considered positive.

### Statistical analyses

Chi square tests and multivariate logistic regression analysis were used to examine the relationships between ACPA, IgM RF, HLA-DRB1 shared epitope and cigarette smoking. Analyses were adjusted for age, sex and disease duration where appropriate. Mann-Whitney U testing was performed to analyse differences in RF titre. Given the heterogeneity between different cohorts, no pooled analysis was made.

All analyses were carried out using the Number Cruncher Statistical System for Windows (NCSS60).

## Results

### Cohort 1

Twenty four percent (141/598) of patients were RF negative. The proportion of RF negative patients did not differ significantly with gender, with 75/300 (25%) females and 66/298 (22%) males testing negative. A weakly positive RF was observed in 77/300 (26%) females and 70/298 (23%) males, while a strongly positive RF was observed in 148/300 (49%) females and 162/298 (54%) males.

Seventy one percent (425/598) of patients were ACPA positive, with 393/425 (93%) demonstrating strong positivity as per ACR/EULAR criteria [4]. There was no significant difference in ACPA positivity between males and females (217/298 (73%) vs. 208/300 (70%), p = 0.32). There was a significant trend of increasing ACPA positivity rates with increasing levels of RF without significant differences between patients who had never or ever smoked (never smoked: 15/71 (21%) RF-ve, vs. 43/64 (67%) RF weak +ve, vs 88/100 (88%) RF strong +ve. Ever smoked: 18/70 (26%) RF-ve vs. 66/83 (80%) RF weak +ve vs.196/210 (93%) RF strong +ve) (Table 1). There was no relationship between ACPA positivity and smoking in patients who were RF negative (OR (95% CI), 1.3 (0.4–5.1)).

Sixty one percent (363/598) of patients had ever smoked, and males were more likely to have ever smoked than females: 214/298 (72%) vs. 149/300 (50%), OR (95% CI) 2.6 (1.8–3.7), p < 0.0001. Smoking >20 pack years was observed in 200/598 (33%), and was significantly different between males and females: 130/298 (44%) vs. 70/300 (23%), OR (95% CI) 2.5 (1.8–3.7), p < 0.0001. Patients with >20 pack years demonstrated significantly increased risk of ACPA positive RA compared to patients who had never smoked (158/200 (79%) vs. 146/235 (62%), OR (95% CI) 2.3(1.5–3.6), p < 0.0001).

There was a significant difference in the proportion of strongly RF positive patients between those who had never smoked (100/235, 43%) and those who had smoked >20 pack years 121/200 (61%), (OR (95% CI), 2.1 (1.4–3.1), p = 0.0002.

No significant differences were seen in ACPA positivity amongst weakly positive RF patients who had never smoked (43/64) and weakly positive RF ever smokers (66/83), irrespective of smoking status or pack years smoked (OR (95% CI), 1.9 (0.8–4.3)).

Strongly RF seropositive never smokers demonstrated ACPA seropositivity predominance (88/100, 88%), as did strongly RF seropositive smokers of >20 pack years (116/121, 96%, Table 3). However, this numerically higher rate of ACPA positivity in strongly RF seropositive >20 pack year smokers is likely to be explained by significantly higher median RF titres of 166 (IQR 92–283), compared to a median RF in never smokers with a strongly positive RF of 115 (IQR 71–226), p = 0.008.

**Table 3:** Cohort 1 relationship between smoking, RF and ACPA.

	Median RF	ACPA neg (%)	ACPA pos (%)	Odds ratio (95% CI)
**Never smoker, RF neg**	neg	56 (79)	15 (21)	1.0 (reference) *
**1–10 PY, RF neg**	neg	13 (87)	2 (13)	0.57 (0.06–3.0) †
**11–20 PY, RF neg**	neg	13 (76)	4 (24)	1.2(0.2–4.5) ‡
**>20 PY, RF neg**	neg	26 (68)	12 (32)	1.7(0.6–4.6) #
**Never smoker, RF weak pos**	22 (17–31)	21 (33)	43 (67)	7.6(3.3–17.9) *
**1–10 PY, RF weak pos**	25 (19–29)	2 (12)	14 (88)	26.1(4.9–249.4) †
**11–20 PY, RF weak pos**	25 (21–31)	4 (15)	22 (85)	20.5(5.6–90.8) ‡
**>20 PY, RF weak pos**	27 (17–30)	11 (27)	30 (73)	10.2(3.8–27.6) #
**Never smoker, RF strong pos**	115 (71–227)	12 (12)	88 (88)	27.4(12.6–59.3) *
**1–10 PY, RF strong pos**	139 (76–277)	5 (13)	34 (87)	25.4(7.8–94.1) †
**11–20 PY, RF strong pos**	153 (67–495)	4 (8)	46 (92)	42.9(12.3–182.0) ‡
**>20 PY, RF strong pos**	166 (92–283)	5 (4)	116 (96)	86.6(27.9–307.6) #

Cochrane- Armitage test for OR trend:

* *P* < 0.0001

† *P* < 0.0001

‡ *P* < 0.0001

# *P* < 0.0001.

**Fig 1:**
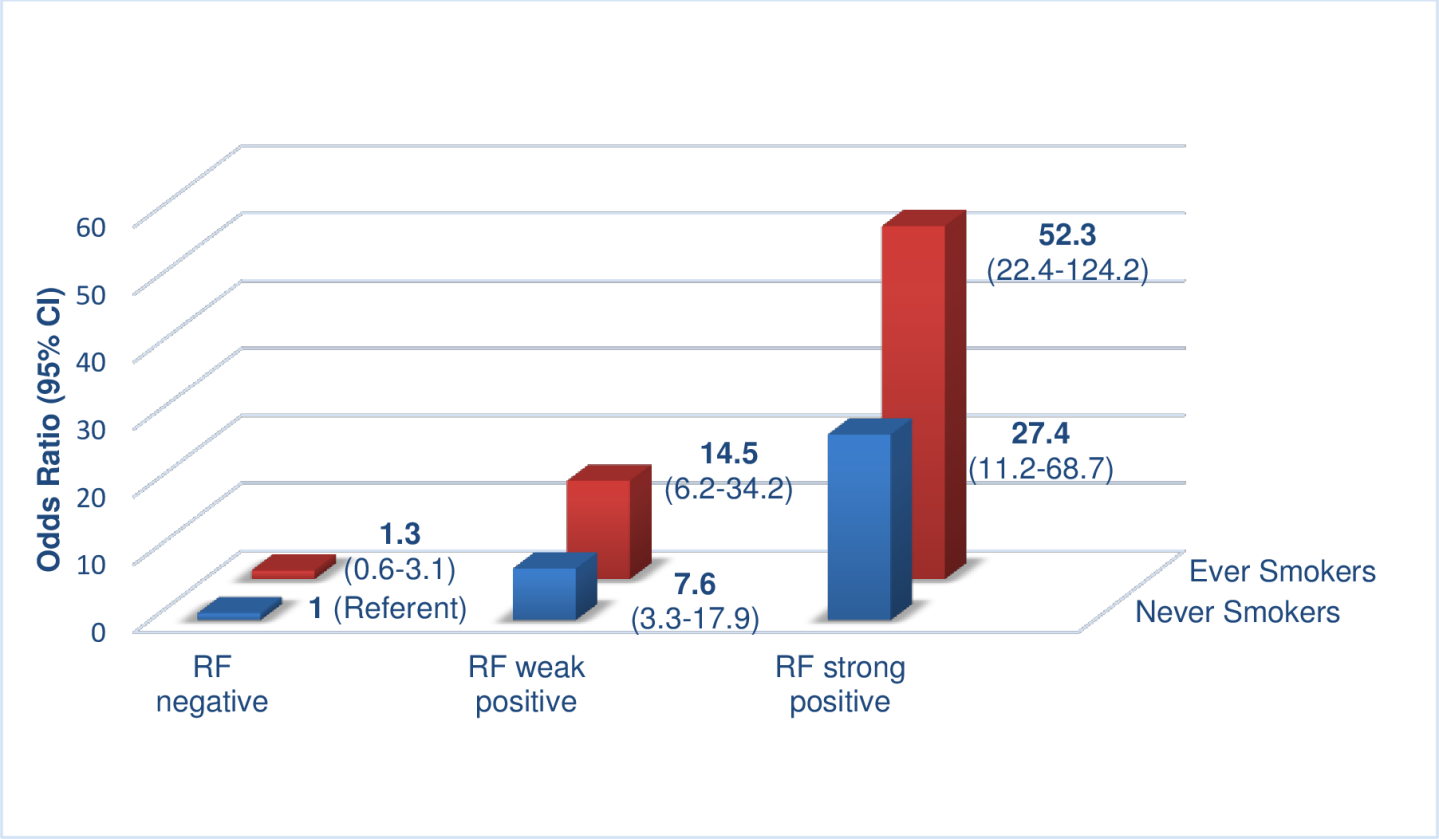
Odds ratio risk of ACPA positivity in RA.

Given the above, and the dominant ACPA positive prevalence (88%) in RF strongly positive never smokers compared to just 32% of RF negative smokers of >20 pack years, odds ratio (OR) risk increases were calculated for both ever smokers (as a combined cohort) and never smokers, with RF negative, weakly positive and strongly positive titres (RF negative never smokers as referent). Amongst ever smokers (n = 365), a stepwise increase in OR is seen, rising from 1.3 (95% CI 0.6–3.1) for RF negative, to 14.5 (95% CI 6.2–34.2) for RF weakly positive, to 52.3 (95% CI 22.4–124.2) for RF strongly positive cases. Whilst not as dramatic, the OR rise for never smokers (n = 235) shows a similar trend; OR 7.6 (95% CI 3.3–17.9) for RF weakly positive, rising to 27.4 (95% CI 11.2–68.7) for strongly positive cases (Fig 1).

From the strength of relationship seen for OR increases with RF titre irrespective of smoking, a further cohort (cohort 2, North Staffordshire) was analysed to determine whether ACPA was determined by increasing titres of RF with reference to SE status.

### Cohort 2

Nearly 40% (162/409) of patients were RF negative in a second cohort of patients in which the SE status had been determined. Seventy five percent (307/409) of patients were ACPA positive (Table 4). There was no significant difference between males and females in the proportion of RF positive or ACPA positive patients. Sixty one percent (235/386) of patients had ever smoked, with a significant difference between males and females (males, 123/150 (82%) vs females, 120/236 (51%), OR 4.3 (95% CI 2.6–7.4), p < 0.0001). As in cohort 1 there was no relationship between ACPA positivity and smoking in RF negative patients (37/74 (50%) never smoked v 45/73 (61.6%) ever smoked; OR 1.6 (95% CI 0.8–3.3).

**Table 4:** Cohort 2 relationship between RF levels, ACPA and shared epitope status (n = 409).

	SE = 0			SE = 1			SE = 2		
	ACPA neg	ACPA pos	OR (95% CI)	ACPA neg	ACPA pos	OR (95% CI)	ACPA neg	ACPA pos	OR (95% CI)
RF	n (%)	n (%)		n (%)	n (%)		n (%)	n (%)	
**Negative**	23 (59.0)	16 (41.0)	**Referent**	29 (43.3)	38 (56.7)	**2.3(1.1–5.6)**	15 (26.8)	41 (73.2)	**4.8(2.0–12.8)**
**Weak +ve**	7(30.4)	16(69.6)	**3.1(1.0–11.4)**	7(11.9)	52(81.1)	**10.0(3.5–33.8)**	5(10.9)	41(89.1)	**10.7(3.4–43.1)**
**Strong +ve**	6(37.5)	10(62.5)	**2.3(0.6–9.4)**	8(13.1)	53(86.9)	**9.0(3.3–28.8)**	2(4.8)	40(95.2)	**23.1(5.5–200)**

We found that the proportion of ACPA positive patients increased with RF positivity in patients carrying 1 or 2 SE alleles but there was only a weak or no relationship in patients lacking a SE allele (Table 4). In RF positive patients the number of copies of the SE (either 1 or 2) was not significant in determining ACPA positivity. However, in RF negative patients, the rate of ACPA positivity appeared to have a greater dependence on number of SE copies, although it did not achieve significance (57% vs 73%, p = 0.06).

Multivariate logistic regression analysis confirmed that RF positivity was strongly associated with anti-CCP positivity (Table 5). Ever having smoked was only weakly associated with ACPA positivity, (Model 1) whereby RF positivity and the presence of the SE were significantly associated. Similar associations were seen in models in which having smoked for >20 years (Model 2) or accumulating >20 pack years (Model 3) were compared with never having smoked, though the association with ACPA was not significant in Model 3.

**Table 5:** Cohort 2 multivariate logistic regression analysis of variables associated with anti-CCP positivity in RA (n = 386).

	Model 1			Model 2			Model 3	
	OR (95% CI)	P		OR (95% CI)	P		OR (95% CI)	P
**Age**	1.00 (0.98–1.02)	NS	**Age**	1.00 (0.98–1.02)	NS	**Age**	1.00 (0.98–1.02)	NS
**Male sex**	1.32 (0.74–2.34)	NS	**Male sex**	1.72 (0.80–3.68)	NS	**Male sex**	2.32 (1.05–5.12)	0.04
**Duration (per yr)**	1.03 (0.98–1.07)	NS	**Duration (per yr)**	1.03 (0.98–1.07)	NS	**Duration (per yr)**	1.03 (0.98–1.07)	NS
**Shared epitope**	3.00 (1.67–5.37)	0.0002	**Shared epitope**	2.26 (1.06–4.79)	0.03	**Shared epitope**	2.45 (1.16–5.17)	0.02
**RF**	4.23 (2.54–7.06)	<0.0001	**RF**	6.09 (3.21–11.54)	<0.0001	**RF**	4.41 (2.35–8.25)	<0.0001
**Ever smoked**	1.73 (1.01–2.94)	0.04	**Years smoked >20**	2.31 (1.15–4.68)	0.02	**Pack years > 20**	1.83 (0.90–3.69)	0.09

## Discussion

We have noted several interesting observations on the determination of ACPA positive RA. Smoking was associated with ACPA positivity only in RF positive patients, and especially in patients who were strongly RF positive. Furthermore, there was a strong relationship between increasing RF levels and ACPA positivity in RA, irrespective of smoking (Table 2). Underpinning this relationship is carriage of the SE. In RA patients who did not carry the SE, we found only weak, or no relationship between RF levels and the frequency of ACPA positivity. This suggests that the co-existence of 2 RA-associated autoantibodies cannot be explained by a propensity for antibody production *per se* as the frequency of ACPA positivity in patients without the SE who were RF negative was not significantly different to those who were RF strongly positive.

There appeared to be a difference in the importance of the number of SE alleles between RF negative and RF positive patients. In those patients who were weakly or strongly RF positive, the number of copies of the SE (either 1 or 2) was not significant in determining ACPA positivity. However, in RF negative patients, the rate of ACPA positivity appeared to have a greater dependence on number of SE copies. If larger studies confirm a significant difference this may pave the way for investigating distinct SE-associated mechanisms giving rise to a positive ACPA in patients positive or negative for RF.

The modest relationship between smoking and ACPA positivity appeared to be due to an increased propensity for smokers to be RF positive. In logistic regression analysis, one model suggested that smoking may be an independent risk factor for ACPA positivity in RA. However, this relationship was far less apparent than the dominant relationship between RF positivity and ACPA positivity. Smoking appeared to demonstrate a significant relationship between the development of both RF and ACPA in the patients studied. There was a clear relationship between smoking in terms of pack years and RF, and ACPA positivity in RA. There did not appear to be a relationship between smoking in terms of pack years smoked and positivity for ACPA alone. Significantly fewer patients with >20 pack years and weakly positive RF were ACPA positive compared to never smokers with positive RF of any titre, further strengthening the argument that the principal determinants of ACPA positivity are RF and SE carriage rather than smoking per se. However, these sub-groups were relatively small and larger studies are required to investigate these sub-groups further.

We excluded 58/658 patients who had ceased smoking >20 years prior to RA onset. Previous studies have demonstrated no increased risk of RA in individuals who have stopped smoking before this time point [11,12]. This cohort demonstrated similar seropositivity trends to RA patients who had never smoked (data not shown). We excluded these patients so that the calculated association between smoking and ACPA positivity was not under represented.

It is noteworthy that the prevalence of RF negative RA was low in cohort 1 (24%). Cornwall has been noted as the poorest region of England [13] and the low prevalence of RF negative RA may reflect a high proportion of socially deprived individuals in this cohort; the majority of the patients were in the most deprived 40% of UK society as 180/300 (60%) females and 173/298 (58%) males had an IMD decile score of 4 or lower. RF positivity associates with social deprivation in other European RA populations [14,15].

This study does have limitations due to its cross-sectional nature. Different classification criteria were used for each cohort. However, as both cohorts are of patients with established RA (median disease duration for cohort 1 = 7yrs, cohort 2 = 9yrs), we do not feel that utilisation of the 1987 RA criteria for cohort 2 and the 2010 criteria for cohort 1 of importance as it is well established that both classification criteria identify patients with established RA. The 2010 classification criteria identifies patients with RA at an earlier time point than the 1987 criteria, however at 5 years of disease duration the number of patients identified by either criteria are the same [16].

Patient notes were analysed to ascertain RF levels at the time of diagnosis in cohort 1. There is the potential for RF titres to vary with disease duration and treatment. A study of 640 inflammatory polyarthritis patients from the Norfolk Arthritis Register demonstrated that over a five-year period, RF status changed in 17% of patients [17]. Critically, this change in RF status was strongly linked to baseline anti-CCP status in this study. The status of RF was 8 times more likely to change in patients with a discordant baseline autoantibody status (RF+/ACPA- or RF-/ACPA+) than in those who were either double negative or double positive (OR 8.2; 95% CI, 4.9 to 13.7; p<0.00001). Thus, RF-negative individuals who seroconverted to RF positive were more likely to be ACPA positive at baseline than were those who remained RF negative (OR, 7.6; 95% CI, 3.8 to 15.3); similarly, those who converted from RF positive to RF negative were more likely to be ACPA negative at baseline than were those who remained RF positive (OR, 7.4; 95% CI, 3.5 to 15.6). Ergo, we feel our data has the potential to have underestimated the relationship between RF and ACPA as the RF test was only undertaken once at the time of diagnosis as per usual clinical practice. ACPA positivity in the same study remained remarkably constant over 5 years of follow up, with only 2% of ACPA negative patients seroconverting to positive, and 4.6% ACPA positive individuals becoming negative between baseline and 5 years. We therefore believe that the cross-sectional nature of ACPA testing would not significantly influence the findings reported here. The authors suggested that repeated testing of ACPA or RF in inflammatory arthritis patients should not be undertaken in routine clinical practice, which also reflects our clinical practice.

Established literature suggests that therapies that target the adaptive immune response, such as rituximab and abatacept, can significantly reduce anti-CCP2 IgG levels [18]. No such reductions in anti-CCP2 IgG levels have been noted in patients treated with methotrexate, tumour necrosis factor inhibitors or tocilizumab. In cohort 1, 18/598 (3%) had an ACPA of below 140 U/ml, with a theoretical possibility of changing from a strongly positive to a weakly positive or negative ACPA through treatment with rituximab or abatacept. However, only 5/598 (0.8%) patients had an ACPA test after treatment with such agents. Whilst we acknowledge that treatment in a cross-sectional cohort may lead to possible misclassification bias due to the mechanism of action of specific disease modifying therapies, the numbers affected in this study are insignificant.

We feel these results can be reconciled with a landmark Swedish study identifying smoking and carriage of the SE alleles as risk factors for ACPA positive RA [1]. In this study carriage of the SE was significantly associated with ACPA positive/ RF negative RA rather than RF positive/ ACPA negative RA. Consequently, the authors suggested that the primary process in RA involved anticitrulline immunity influenced by an interaction between smoking and carriage of the SE. However, in this study only a very small proportion of the ACPA positive RA cases were RF negative (13%) and the clear majority (87%) were double positive for both RA autoantibodies (far more than would be expected by chance alone). Ergo, given the overall strong association between the carriage of SE alleles and ACPA positivity it follows that the SE is principally associated with ACPA positive / RF positive RA. Likewise, the observation that smoking interacts with the SE to generate ACPA positivity overall in RA is factually correct; more precisely this interaction is between smoking, the SE and both RA autoantibodies. This has recently been proven to be the case as pooled analysis of 2234 RA patients has suggested that smoking interacts with the SE to generate multiple RA associated antibodies rather than ACPA alone [6].

We have suggested that the citrullinated heavy chain of immunoglobulin G (IgG) is the dominant antigen in RA and generated by B cells in the lungs of smokers; a process facilitated by SE alleles with the B cell acting as both an antigen presenting cell and the source of the antigen [19]. This process need not be exclusive to the lung as the rheumatoid joint could be the host for B cell activation and therefore could theoretically occur in both smokers and never smokers.

Citrullination of the Fc region of IgG may generate an antibody response specific to RA, consequently generating both a strongly positive RF and ACPA response. This hypothesis is supported by the recent finding of citrullinated IgG in the synovial tissues of RA patients, with IgG as a target of peptidylarginine deiminase (PAD) 2 and PAD4 activity in RA synovial biopsy tissues [20]. Another recent study observed over 80 different citrullinated peptides in the RA synovial fluid including the heavy chain of IgG. [21]. Furthermore, the demonstration of bispecific antibodies against cyclic citrullinated peptides and IgG in RA suggests that citrullinated IgG may generate both a RF and ACPA response [22].

We do not consider that ACPA positivity is likely to have given rise to a subsequent RF response in the RA patients studied here as a study of the RF isotypes (IgM, IgG and IgA) in individuals who subsequently developed RA were more likely to be present before the development of various ACPAs (fibrinogen, α-enolase, triple helical collagen type II, filaggrin and vimentin). Previous data suggests the frequency of ever being positive for IgM-RF in individuals who developed RA, but were negative for the above ACPA was 16–31% [23].

Recent analysis of specific immunoglobulin subtypes to ACPA, specifically IgG types 1–4 to anti-CCP, has been demonstrated [24]. It has been recently suggested that IgG1 and IgG4 anti-CCP antibodies associate with the shared epitope, and IgG2 associates with smoking. However, low patient numbers in analysis of individual subtype classes limit the applicability of this data. Furthermore, other evidence suggests that the shared epitope has been associated with elevated IgG3 and IgG2 ACPA titres. Selective support of the Th1 response among RA patients homozygous for HLA-DRB1*04 has been suggested in IgG3 subtypes [25].

Our study does not aim to address specific IgG subclasses [24] and IgA responses [26] to ACPA. This would be worthy of further study taking into account RF levels at diagnosis, and the smoking history of patients.

## Conclusions

These results raise important questions about the relationship between RF and ACPA in RA and the potential mechanisms underlying this relationship. We feel that processes involving the simultaneous or sequential generation of both RF and ACPA need to be considered in further studies investigating RA pathogenesis irrespective of smoking.
